# Salivary Factors that Maintain the Normal Oral Commensal Microflora

**DOI:** 10.1177/0022034520915486

**Published:** 2020-04-13

**Authors:** G.H. Carpenter

**Affiliations:** 1 Salivary Research, Centre for Host-microbiome Interactions, Faculty of Dental, Oral & Craniofacial Sciences, King’s College London, London, UK

**Keywords:** metabolomics, acetate, bacteria, secretory IgA, mucosal pellicle, resilience

## Abstract

The oral microbiome is one of the most stable ecosystems in the body and yet the reasons for this are still unclear. As well as being stable, it is also highly diverse which can be ascribed to the variety of niches available in the mouth. Previous studies have focused on the microflora in disease—either caries or periodontitis—and only recently have they considered factors that maintain the normal microflora. This has led to the perception that the microflora proliferate in nutrient-rich periods during oral processing of foods and drinks and starves in between times. In this review, evidence is presented which shows that the normal flora are maintained on a diet of salivary factors including urea, lactate, and salivary protein degradation. These factors are actively secreted by salivary glands which suggests these factors are important in maintaining normal commensals in the mouth. In addition, the immobilization of SIgA in the mucosal pellicle indicates a mechanism to retain certain bacteria that does not rely on the bacterial-centric mechanisms such as adhesins. By examining the salivary metabolome, it is clear that protein degradation is a key nutrient and the availability of free amino acids increases resistance to environmental stresses.

## Introduction

The common perception of bacteria in the mouth is that they reside there because of the available warmth, moisture, and protection and they take advantage of the regular input of nutrients from food whilst providing little or no benefit to the host. At best their contribution to oral health appears to be exclusion of pathogenic bacteria by maintaining a commensal population of bacteria and fungi. Possibly this view of oral microbes has been driven by research investigating the causes of dental caries. However, more recent studies have been examining the oral microbiome in normal healthy (caries free/treated caries) subjects (Zaura et al. 2017), the influence of non-sugar aspects of diet (De Filippis et al. 2014), and the other nutritional sources (Jakubovics 2015; Gardner et al. 2019) which paint a different picture in which the host actively promotes the growth of certain bacteria by providing them with suitable nutrients to maintain growth. A major benefit of the oral microbiome to whole body physiology has already been described—the nitrite-producing bacteria on the tongue which contribute to nitric oxide production and the lowering of blood pressure (Webb et al. 2008). There are likely to be others as more studies explore the oral metabolome in relation to whole body health. Clearly, if there is a benefit to whole body health then the body should nurture the oral microbiome. If true, this could explain the recent concept of “resilience” (Rosier et al. 2018), the ability of the oral microbiome to resist pressure to change from antibiotic treatment or overgrowth of one species, into a dysbiotic state often associated with disease. Crucial to the process of maintaining oral commensals is saliva. Previously, most studies have described the anti-microbial properties of saliva as bacteriostatic with some bacteriocidal properties, which it clearly has, but this paper will also review the evidence that it has bacterial growth–promoting properties as well. Broadly speaking, the growth-promoting properties can be split into three main sections; nutrients, attachment, and environment.

## Nutrients

**Figure 1.**
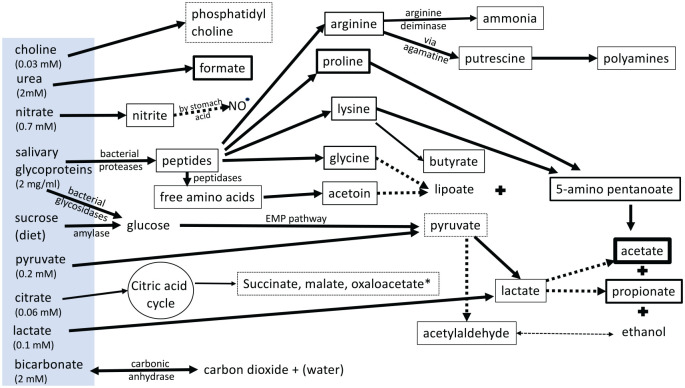
The main bacterial substrates (blue box) and detected metabolites (indicated by boxes) in whole mouth saliva. The thickness of arrows and boxes indicates relative abundance, dotted lines indicate possible connections. Under resting conditions between meals, the products of the citric acid cycle (indicated by *) are largely undetectable. Most metabolites indicate the breakdown of salivary glycoproteins as the main nutrient source, the amino acids yielding acetate and propionate, the N- and O-linked glycans leading to pyruvate via the Embden Meyerhof Parnas (EMP) pathway.

**Figure 2.**
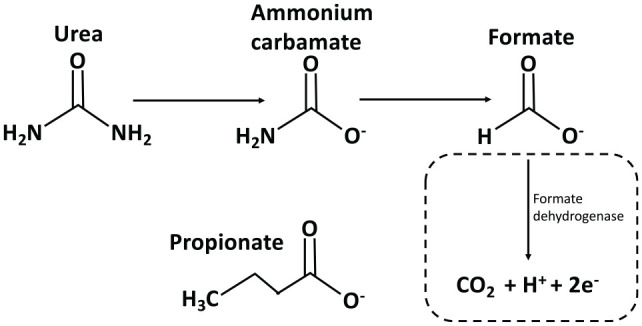
C^13^ labeled urea was added to whole mouth saliva and incubated for 1 h at 37°C. C^13^ nuclear magnetic resonance analysis revealed peaks assigned to ammonium carbamate and formate. In addition, propionate and acetate were detected of which only acetate was detected in the unlabeled control sample due to the natural abundance of C^13^ acetate isoform. The presence of ammonium carbamate and formate suggests urease is not active in reducing urea to ammonia. It is unclear how labeled propionate appeared or why formate is not further reduced to carbon dioxide by formate dehydrogenase (dotted box).

Most of the nutrients for oral bacteria are specifically added and are not merely leakage from the serum compartment. Saliva is formed by an active process of ion secretion into the lumen of the gland, creating an osmotic gradient (Thaysen et al. 1954) which draws water through from the interstitial space. Most ions and metabolites are transported by specific channels into saliva. Proteins are synthesized in the glands and added mostly by a separate mechanism of storage granule release dependant on cyclic adenosine monophosphate (AMP) signaling (Castle and Castle 1998) and as a consequence few serum proteins are found in saliva collected directly from the duct. In contrast, whole mouth saliva contains some serum proteins derived from a serum transudate leaking around teeth (via gingival crevicular fluid). In a recent comparison of metabolites in parotid saliva, whole mouth saliva and plasma (Gardner et al. 2019) urea concentrations were greater in parotid saliva than whole mouth saliva or plasma implying the active transport of urea into parotid saliva, presumably by the urea transporters (UT-A and UT-B) although their presence hasn’t been confirmed in salivary glands so far. In our study, urea was one of the few components to decrease in whole mouth saliva relative to parotid saliva suggesting its uptake and use by bacteria. Urea is the most abundant (non-protein) nutrient in saliva (Fig. 1) used by bacteria such as *Streptococcus salivarius, Actinomyces naeslundii*, and *haemophilus* apparently through their expression of urease (Chen et al. 1996), an enzyme that converts urea to ammonia and carbon dioxide. Whilst the production of ammonia in plaque would help to neutralize lactic acid in caries lesions (Gordan et al. 2010), a recent review concluded there was no beneficial effect on caries (Zaura and Twetman 2019). To further understand the metabolism of urea by oral bacteria C^13^ labeled urea was added to an expectorated whole mouth saliva sample and incubated for 1 h (Carpenter *unpublished data*). The sample was then analysed by C^13^ nuclear magnetic resonance (NMR) which permits the tracking of the added urea. Surprisingly, urea was seen to be first converted into ammonium carbamate and then to formate and propionate (see Fig. 2 and Appendix 1 for spectra). Although conversion of urea to ammonium carbamate has been described before, even by urease (Mobley et al. 1995), it is then assumed to degrade into ammonia and carbon dioxide. Indeed, this reaction is so reliable that it is the basis of the urea breath test for *Helicobacter pylori* infections of the gut (Megraud and Lehours 2007). If urease activity was present in the mouth this would compromise the urea breath test. A more logical explanation is that the ammonium carbamate is converted to formate and not ammonia. This is interesting as it could account for the large amounts of formate in saliva and the lack of efficacy of urea in preventing caries. The present results do not exclude the possibility of urease action and whether ammonia is produced may depend on the amount of urea added. Clearly more work is required to substantiate this new idea and delineate which bacteria convert urea to formate and/or which convert to ammonia.

Resting whole mouth saliva, which is present when there is no food in the mouth, has very low levels of sugars/carbohydrates present. Typically, parotid saliva has around 20 to 100 umol/l glucose (Andersson et al. 1998), but the glucose becomes undetectable in resting whole mouth saliva, presumably because the bacteria rapidly utilize it via the Embden Meyerhof Parnas (EP) pathway (Fig. 1). The greatest sources of carbohydrate are food itself, which can still be detected in saliva 20 min after consumption although it is usually cleared from the mouth after 1 h. Thus, most of the time bacteria in the mouth are utilizing intrinsic nutrients in saliva as their substrates (Jakubovics 2015). So if the commensal bacteria are not utilizing glucose to any great extent, what nutrients do they use? The metabolomic analysis of whole mouth saliva indicates the proteolytic degradation of salivary proteins fuels many bacteria (Fig. 1). The abundance of free amino acids in whole mouth saliva (Syrjanen et al. 1990) contrasts with their almost complete absence in sterile saliva collected from the gland (Gardner et al. 2019). Their degradation via 5 amino pentanoate to acetate and proprionate (Cleaver et al. 2019) probably accounts for the most abundant metabolites in saliva. Although some amino acids, such as proline, appear not to be utilized as it is one of the most abundant in saliva (Santos et al. 2020), lysine, glycine, glutamate, and arginine are further utilized. The Arginine Deiminase System (ADS) hydrolyses arginine to create citrulline and ammonia; the ammonia is beneficial to the host by neutralizing lactic acid in carious lesions. This pathway has become prominent as some dental products now contain arginine as an additive. A recent study found a reduction in sucrose metabolism when subjects used an arginine-containing toothpaste which was associated with altered salivary microflora, but not altered plaque (Koopman et al. 2017). Although arginine is being added to toothpastes, it’s interesting to note that saliva already contains many free amino acids, including arginine (Syrjanen et al. 1990) from proteolysis of salivary and cellular proteins by bacterial and mammalian proteases (Vitorino et al. 2009).

In addition to the amino acids, the sugars linked to the proteins are also utilized; many bacteria contain sialidases (McDonald et al. 2016) and other glycosidases which can be utilized by the glycolytic EMP pathway to form pyruvate and formate. The close association of bacteria in biofilms permits the complete degradation of salivary glycoproteins as no single bacterium contains all the necessary enzymes (Wickström et al. 2009). Mucins are often cited as being important nutritional additives for oral bacterial culture systems, presumably due to their high sugar content, but in fact, most salivary proteins are glycosylated to some degree (Carpenter et al. 1996) and indeed the basic proline-rich proteins, agglutinin and SIgA have the same O-linked glycans as mucins (Cross and Ruhl 2018).

The active secretion of nutrients into saliva is perhaps the best evidence of positive selection of microbes in the mouth and the best characterized is the nitrate/nitrite system (Hezel and Weitzberg 2015). In this system, salivary glands actively transport nitrate from the blood system, via the sialin transporter and deliver it into saliva (Qu et al. 2016). Bacteria including *Rothia* and *Veillonella* within the mouth then convert the nitrate to nitrite which can be converted to nitric oxide when the nitrite reaches the acidity of the stomach. Several studies have shown salivary nitrate to correlate to lowered caries risk (Doel et al. 2004) and longer supplementation with nitrate appeared to alter the microbiome suggesting some degree of utilization (Burleigh et al. 2019). Other important nutrients include lactate, bicarbonate, and vitamins. The role of lactate appears central to the food networks that permit the high diversity of bacteria in the mouth (Jakubovics 2015). Food networks describe how lactate producers co-exist with lactate consumers in multi-species biofilms thus permitting a larger variety of bacteria to co-exist through beneficial exchange. In low sugar/carbohydrate environments most lactate is delivered by saliva derived from plasma—the active salivary gland secretion of lactate (as opposed to leakage) again suggesting the host selection of bacteria. Bicarbonate is another essential nutrient used by many bacteria such as *Streptococcus anginosus* (Matsumoto et al. 2019) and *Porphyromonas gingivalis* (Supuran and Capasso 2017), but it is actively secreted by salivary glands as part of the fluid secretion mechanism particularly for mucin-secreting sublingual and minor glands (Lee et al. 2012). Bicarbonate could also form a food network although it is less well studied than lactate. Some bacteria are bicarbonate consumers whereas some are bicarbonate producers. *P. gingivalis* expresses carbonic anhydrase which forms bicarbonate by the hydrolysis of carbon dioxide (Supuran and Capasso 2017). As well as propagating certain bacteria by supplying certain nutrients, saliva also limits the availability of other key nutrients. For example, there are very low levels of cobalamin (vitamin B12) in saliva which are an important nutrient for some bacteria, particularly *P. gingivalis*. As well as not transporting any into saliva from serum, saliva also contains vitamin-binding proteins such as transcobalamin which strongly binds cobalamin and prevents its use by bacteria. This chelation of nutrients is similar to lactoferrin for iron or haem. Salivary glands secrete iron-free lactoferrin which avidly binds iron and thus prevents bacteria utilisation. This could be interpreted as the body wishing to keep the certain bacteria quiescent and is an important mechanism in resilience. As shown by oral diseases, the availability of alternative nutrient sources such as serum (for periodontitis) or plant-based sugars (for caries) encourages pathogenic traits in bacteria. Overall, the nutrient needs of bacteria are varied but can be completely supplied by saliva but only by the coordinated actions of bacteria. If most of the nutrient needs can be supplied by saliva through mostly proteolytic degradation, a central question is why are there so many saccharolytic bacteria in the mouth—one possible explanation is specific attachment.

## Attachment

**Figure 3.**
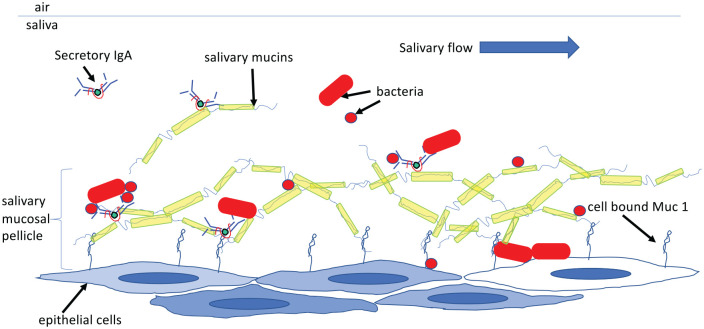
Secretory IgA (SIgA) complexes with salivary mucins (Muc 5B and Muc 7) before binding to epithelial membrane–bound mucin Muc 1 to form the salivary mucosal pellicle. Secretory IgA can then mediate binding of bacteria (red rods and circles) helping them to adhere to epithelial cells. The mucin hydrogel–like properties of the mucosal pellicle allow concentration of bacterial products allowing quorum sensing and food networks that enhance their growth. As the epithelial surface is constantly sloughing, thick biofilms do not occur as they do in plaque around teeth. (Not drawn to scale).

Most research concerning attachment has focused on mechanisms by which bacteria bind teeth to understand the dental caries process. Selected salivary proteins bind the enamel surfaces forming what is termed the “acquired enamel pellicle” the bacteria then bind these proteins through adhesins expressed on pili projecting from the surface of the bacteria (Cross and Ruhl 2018). The adhesins are usually lectin-like molecules which bind the glycans attached to the salivary proteins (Bensing et al. 2016). These glycans are either N- or O-linked to the peptide backbone and often terminate in sialic acid. Several bacteria make sialidases to remove and utilize sialic acid (McDonald et al. 2016) and to gain access to galactose, fucose, and mannose glycans for either nutrition or attachment (Wong et al. 2018). Although there is some specificity of bacteria for certain glycans, many salivary proteins express the same glycans. For example, the Tn antigen (GalB1-3GalNAc) is an O-glycan frequently found on the salivary mucins MUC5B and MUC7 (Chaudhury et al. 2016), but this antigen is also present on many of the basic proline-rich proteins (Carpenter and Proctor 1999) which are the most abundant group of proteins in parotid saliva. Most previous research gives the impression that it is the bacteria which are binding to the salivary proteins to prevent being swept away by the nearly constant flow of saliva. But is there any evidence that salivary proteins actively promote the adherence of specific bacteria? One possible mechanism involves secretory IgA (SIgA). This antibody is often cited as preventing colonization as it agglutinates bacteria in solution due to its dimeric arrangement (Fig. 3). Recently it has been shown that SIgA also forms part of the mucus attached to mucosal surfaces of the mouth (mucosal pellicle) (Gibbins et al. 2014). The SIgA binds the salivary mucins (Biesbrock et al. 1991) via mucin-mucin interactions (Gibbins et al. 2015) in solution and then binds the cell membrane mucin MUC1 (Ployon et al. 2016). Even though not all of the salivary SIgA binds to the mucosa it does concentrate to high levels forming an immune reservoir. By doing so, SIgA would aid colonization of mucosal surfaces by bacteria that SIgA is reactive against. It is known that SIgA binds many oral bacteria, such as *S. mitis, S. oralis*, and *S. mutans* using shared epitopes (Cole et al. 1999). A role of mucosal bound SIgA determining commensal bacteria has been demonstrated in the gut (Donaldson et al. 2018). It’s possible then that SIgA influences which bacteria are present in the mouth by specifically binding them. This would be particularly important for the Streptococcal species that grow best in high sugar environments. It would interesting to investigate if bacteria bind epithelial cells in babies since at birth SIgA is relatively scarce (Seidel et al. 2001) but develops over the first year with increased exposure to bacteria. In general, there are no changes in SIgA availability or epitope recognition with ageing.

## Environment

The mouth has the greatest variety of bacteria compared to other sites of the body probably because of the variety of niches available. Most of the mucosal and dental surfaces will be covered in bacteria fed by saliva and occasional nutrients from foods using aerobic respiration. Using an in vitro model (saliva inoculated hydroxyapatite discs, cultured in sterilised saliva) aerobic conditions mimicked salivary metabolites with acetate, proprionate, and formate being the most abundant metabolites. Whereas the same cultures under anaerobic conditions led to a loss of glycine and lactate production and an increase in ethanol production (Cleaver et al. 2019). In addition to the aerobic sites there are a number of anaerobic sites: in crevices on the tongue supplied by saliva or within plaque, either supra-gingival, fed by saliva, or sub-gingival pockets fed by the gingival crevicular fluid, a serum filtrate. Salivary metabolomics is dominated by the aerobic metabolism of streptococci degrading salivary glycoproteins except when sugars become abundant following ingestion of food. In contrast, tongue and dental plaque metabolomics indicate anaerobic activity, particularly when protected within a biofilm structure. Each of these sites will have very different metabolic and genetic composition. Presumably the body would prefer most bacteria to remain aerobic as most disease is associated with anaerobic biofilms. Although saliva does contain many anti-bacterial proteins and enzyme systems (peroxidase) it is not as anti-bacterial as other sites such as the eye or the lungs (Lloyd-Price et al. 2016) which again supports the concept that the body propagates the oral microbiome rather than opposing colonization.

One aspect of environment that has not been studied extensively is the effect of age. Several factors affect the supply of nutrients to the mouth as already outlined in Gardner et al. 2018. The amount of exercise, nutrition, and dental status will affect the salivary metabolome. In addition, the number of pockets or crevices on the tongue and around teeth will increase with age. A large study indicated several metabolomic changes with increasing periodontal disease (Liebsch et al. 2019), most notably the increased production of phenylacetate. Salivary amino acids have also been shown to alter with ageing (Tanaka et al. 2010). All these changes are likely to change many aspects of the salivary metabolome but at present not so many studies have been completed on healthy individuals whilst controlling for all the variables listed above that may confound the results.

## Discussion

If the body does promote the colonization of the mouth by any of the mechanisms outlined above, this would increase the resilience of oral bacteria by providing alternative sources of nutrition and increased residence time in the mouth. The conversion of urea to formate and propionate would allow the bacteria to extract energy from the process whereas no adenosine triphosphate (ATP) is formed during the conversion of urea to ammonia and carbon dioxide (Burne and Marquis 2000). In addition, the presence of free amino acids in saliva also increases the ability to resist stresses (osmotic, smoking, and heat) in solution since many amino acids can buffer pH, osmotic, and redox changes by themselves. But some amino acids, if taken up by the bacteria, also confer resistance to osmotic and oxidative stress (Christgen and Becker 2019). The ability of the microflora to recover from antibiotic use is a hallmark of a healthy oral microbiome. So could these mechanisms be used when a dysbiotic state exists in the mouth? Most of the ideas outlined are only relevant to mucosal-bound bacteria. As a group they are distinct to other sites in the mouth (O’Donnell et al. 2015) but may equal the number of bacteria present in plaque. These mechanisms are unlikely to apply to the pathogenic bacteria on or around teeth because these are special niches fed by different nutrient sources (serum or diet) often protected from saliva by extracellular matrices or by existing within pockets. Presumably removing this nutrient source should reduce the pathogenic bacteria which is easier to achieve for diet-related but not for serum-fed biofilms. It seems unlikely that the nutrients identified in Figure 1 would affect plaque microbiology as shown by an arginine supplementation study which altered the salivary microbiome and metabolome but not the plaque microbiome (Koopman et al. 2017). Another interesting implication is that these prebiotics could affect taste. Arginine supplementation has been shown to affect taste (Melis and Barbarossa 2017), dietary protein associated with differences in the oral microbiome (De Filippis et al. 2014), and bacteria derived D and L amino acids are known to bind taste receptors (Lee et al. 2017). As protein degradation accounts for most of the metabolites found in resting whole mouth saliva it suggests it is an important factor in determining the composition of the oral flora. Factors involved in this process may be useful prebiotics. Based on Figure 1, one obvious but missing factor is lipoate. Lipoic acid is an essential co-factor for several of the amino acid pathways and is pivotal to the virulence of *Staphylococcus aureus* (Zorzoli et al. 2016), but despite having a clear NMR signature it could not be detected in any samples in our studies. Its absence may suggest it is the rate-limiting step in many bacteria and thus may form a useful prebiotic to alter a dysbiotic microbiome back toward normal metabolism. Interestingly, lipoic acid as an oral treatment for burning mouth (Femiano and Scully 2002) has had some success although no studies to date have examined changes to the oral microbiome.

In summary, the nutrient supply by saliva suggests a deliberate attempt to maintain certain bacteria and exclude others. This propagation is aided by the selective absorption of bacteria onto mucosal surfaces by the immobilization of SIgA into the mucosal pellicle.

## Author Contributions

G.H. Carpenter, contributed to conception, design, and data analysis, drafted the manuscript. The author gave final approval and agrees to be accountable for all aspects of the work.

## Supplemental Material

DS_10.1177_0022034520915486 – Supplemental material for Salivary Factors that Maintain the Normal Oral Commensal Microflora Click here for additional data file. Supplemental material, DS_10.1177_0022034520915486 for Salivary Factors that Maintain the Normal Oral Commensal Microflora by G.H. Carpenter in Journal of Dental Research
