# Supplementary material for: Salivary Factors that Maintain the Normal Oral Commensal Microflora
Source: J Dent Res. 2020 Apr 13;99(6):644–9. doi: 10.1177/0022034520915486 (PMC7536522; doi:10.1177/0022034520915486)
Supplement: DS_10.1177_0022034520915486 – Supplemental material for Salivary Factors that Maintain the Normal Oral Commensal Microflora [file DS_10.1177_0022034520915486.pdf]

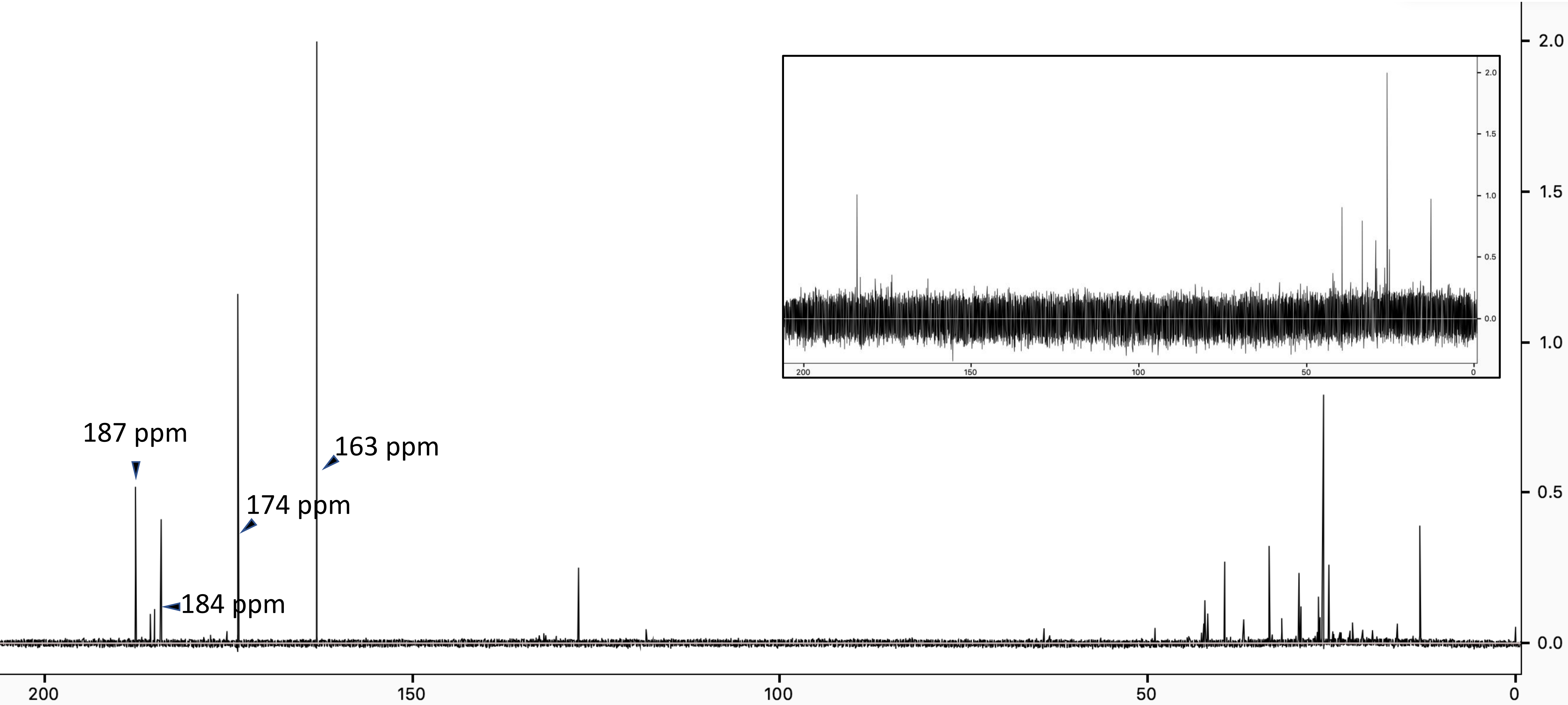

Appendix 1. Carbon 13 spectra of saliva sample incubated with 10 mM C13 labelled urea and incubated for 24 hours at 37 degrees C. Peaks at 174 ppm relate to formate, 163 ppm indicate a quarternary carbon possible ammonium carbamate, 187 ppm indicates propionate and 184 ppm indicates acetate. Only the peak at 184 ppm appeared in the control (no added C13 urea) saliva sample (see inset).
